# Supplementary material for: Patterns of Intron Gain and Loss in Fungi
Source: PLoS Biol. 2004 Nov 30;2(12):e422. doi: 10.1371/journal.pbio.0020422 (PMC532390; doi:10.1371/journal.pbio.0020422)
Supplement: Table S1 — Also available at http://genes.mit.edu/NielsenEtAl/. (4.3 MB ZIP). [file pbio.0020422.st001.zip › NielsenEtAl/html/450.html]

AN2868.1.NCU04761.1.MG02478.1.FG08476.1


```
 CLUSTAL W (1.82) Multiple Sequence Alignments - Introns Inserted


Sequence 1: MG02478.1	575 aa
Sequence 2: FG08476.1	576 aa
Sequence 3: NCU04761.1	568 aa
Sequence 4: AN2868.1	802 aa
Alignment Length: 827 aa
Number Identitical Residues: 235 aa
Alignment Score (without introns) 11115


MG02478.1 	MPCFKGIAVSIHAN-GPIPEHGIVKQSRASRVTSYIPVPEPTLNPETSA-QEPAKFAISI
NCU04761.1	MPCFKGIAVSIHANGAPLPEYGIQKQSRVSRINTYIPVPQPQISPDSGR-PEAAKFAISI
FG08476.1 	MPCFKGIAVSIHANSAPLPEHGMQKQSRLSRISTYIPVPQPQL-AESNK-PEPAKFAISI
AN2868.1  	MPCFKGLAVSIHTPDGPLPEYSIQRQSRASRITSYIPVPPPKIPDTAGGKPEQSTFAISI
          	******:*****: ..*:**:.: :*** **:.:***** * :   :. . * :.*****

MG02478.1 	TLLTKGLQIPYSTPPPTADDPYPKPRIVG----APGQNGS----VSPYIPLTSSENETIA
NCU04761.1	TLLTPGLAIPYSTPKATENNPYPKPAFVGTMPTSTGERGKYHGVVAPYIPMTNSENETIA
FG08476.1 	TLLTPGLPIPYSTPKPSDENPYPKPQFVGGL--NTGERGKSTGVVNPYIPMTNSENETIA
AN2868.1  	TLLNPGQDVPYSAGKPTPDNPHPKPKVVGGLPGQTEQRGLYTSTVAPYQALTNSPNETVA
          	***. *  :***:  .: ::*:*** .**  .  . :.*   . * ** .:*.* ***:*

MG02478.1 	AYIYFDGRAKEEVATLLRPGEETWVNSRWVQVPESEGGGLAEREFLFREVALERWLNGLD
NCU04761.1	AYIYFDGRAKEEVATLLRPGEETWVNSRWVQVPPSEGGGLAEREFLFREVGLERWLNGLD
FG08476.1 	AYIYFDGRAKEEVATLLRPGEETWVNSRWVQVPDAEGGGLAEREFLFREVGLERWLNGLD
AN2868.1  	AYIYFDGRQKEEVATLLRRGEETWVNSRWVSVPESEGGGLAEREFLFREVGLERWLNGLD
          	******** ********* ***********.** :***************.*********

MG02478.1 	LKG-HDAADKLERRKHKYERRQRRQ-----EKAAAAAENG----------GVRSTSPMDS
NCU04761.1	LQG-HDAAEKLERRRQKFEKRRRRQKAISEGNAASGRLQG------TLRYGSEDR-PVEA
FG08476.1 	LQG-HDAAEKLEKRRQKFEKRRRRQGAL-EGNASADIEEGSSSRRSTLRYGADDKSPIEA
AN2868.1  	LEGNKDAAAKIERRRQKMEKRRAKRALDGNNDLDMDDKKRD---KGVMRYGNDTRSPLED
          	*:*.:*** *:*:*::* *:*: ::   .. .      : .    .    *    :*:: 

MG02478.1 	---DSENSSAESDDEIPEAAGQIKVAMFRVLASGEIKKGEYSPQFDAHDDDDDDGAGGNQ
NCU04761.1	V-DEDASLSSSDDDEPPEATGQIKVAMFRVIASGEIKKGEYSPQFDAHDDDDEE--GGSK
FG08476.1 	V-LDDSDSWSDDDDEPPEATGQIKVAMFRVLASGEIKKGEYSPQFDAHDGDEDD--MGSK
AN2868.1  	VSDDDVSLASSDDDPVPESTGQIKVALFRVLASGEIKRGEYSPQFDAHDDDEDG-----Q
          	 : :. .  :..**  **::******:***:******:***********.*::      :

MG02478.1 	NGS-GVGADVEHTTSFAKPKTLDPKTISTQTVTGIDGPDKPYATFTFFYRGKR1QLEKMG
NCU04761.1	SGNNGIDADVEHTTSFAKPKTLDPKTISTQTVTGIDGPEKPYAVFTFFYRGER1QLQKMG
FG08476.1 	EGN-GIDADVEHTTSFAKPKTLDPKTISTQTVTGIDGPDKPYAVFTFFYRGER1QLQKLG
AN2868.1  	GGQGGGDEDIDHTTSFAKPKTLDPKSISTQTVTGIDPSDKPYAIFTFMYRGER1QLQKMG
          	 *..* . *::**************:********** .:**** ***:***:* **:*:*

MG02478.1 	VLPSSKTQATPSSAVKRRSAQIELPKLGPLKTGGTVGFSAFRDNDADTARRRKSRKKSIA
NCU04761.1	VLASEKQQVTSPTAAKRRSAQLDFSSLGPLKKQGTVGFSTFRDRDAEP-RPKKSRKNTNG
FG08476.1 	VIQSSKNPQTTPSSVKRRSGQLDFSNLGPLKAGGTVGFSAFRDQASEASRRRKARKKSNG
AN2868.1  	ILKDSKSQETP-GSAKRRSQQLDFANLGPLKPGGTVGFLNFRDN---TEGRRKSKKDKGV
          	:: ..*   *.  :.**** *:::..*****  *****  ***.   .   :*::*..  

MG02478.1 	GSPSTGVAAAMAAADSDSDADEDSIATKMEDTDEKDVIQTGATLNPDEAKFSRELADGVE
NCU04761.1	KAG-------AMDEDSDDEDDDEDISDKMLADDEDNVDPS--KQGPEDVKFTGELADGVN
FG08476.1 	N---------IADDSDDDDDDDDANLGKMEEAYDKDVDTK---LAPEDAKFQGELADGVD
AN2868.1  	VDD---------DDMDSDDDEDDRILNQADEEEDKEDDQH---LSPDDIRRQGELAEGLR
          	               ...: :::    :     :.:         *:: :   ***:*: 

MG02478.1 	RIH0LKRARSADPDSAAANPVKTENQQPASQSNTPDNKSLLESLSPVPSTS-ATFGGSAF
NCU04761.1	RIK0LKRAHDAGQSSTTDT---------QAGGSAPENGS---APGTTAGLS------SLF
FG08476.1 	RIH0LKRAHSADPDSIAAS--------PASQNNNTVNSS--TSLAPTAGGSPANNSLSNA
AN2868.1  	QIK0LKRQHSAASLAGSSRR-DTASPKTTGATPTSTNTSTTPPAATATSAPTTTGTLEGQ
          	:*: *** :.*   : :    .: . .. .    . * *   . ..... ..:.   .  

MG02478.1 	NNKNIPDDAIVGSPLKKQR--ALSGPDAEKLS-QAAGLGSILAQVQAGQTPPASSGLQPS
NCU04761.1	PKSLTGEAAAVGSPMKKARASVDDVGSDVKPL-GGSSFVPSVPSGPNGILAQAAETPLPQ
FG08476.1 	ASLDTPSKSTFQSPLKKYRPSVDYGADGIIFG-SGSGLKTELDAAPSTGSGPGTPSNAEV
AN2868.1  	STNTLNGEEFLGSPLKKQRPSVSQADESLLRQRMGSGLSQQISDVLGSGAQSGSVTNNSK
          	 .        . **:** *.:.    .       .:.:   :          .:      

MG02478.1 	AMKQEDEEEEL-----------------~-----------------------~-------
NCU04761.1	TMGGEMDEEEL-----------------~-----------------------~-------
FG08476.1 	QKPKIEDEEEL-----------------~-----------------------~-------
AN2868.1  	PPLGPSHDESLQSPKTMNPPDSVDEELY2TTQTTPNMPLTRSTDPLVWIDCE0MTGLDPT
          	      .:*.*.:..: ....: ...   ::.::.. . : ::..    ...  :. ..:

MG02478.1 	------------------------------------------------------------
NCU04761.1	------------------------------------------------------------
FG08476.1 	------------------------------------------------------------
AN2868.1  	TDHILQICCFITDADLNLLDATGFEAVIHVPQSALDSMSQWCIDTHGRTGLTQRVQNSTV
          	:.   . ..  :.:. .  .::. .:    ..:: .: :. . .: . :. :.  ..:: 

MG02478.1 	------------------------------------------------------------
NCU04761.1	------------------------------------------------------------
FG08476.1 	------------------------------------------------------------
AN2868.1  	TPEAAAADLLAYIKRYIPKPRTALLAGNSVHADKAFLACGPYAAILEWLHYRIVDVSTIK
          	:..::::.  :  .   ... ::  :..:  :..:  :... ::  .       . :: .

MG02478.1 	---------------------------------------------------
NCU04761.1	---------------------------------------------------
FG08476.1 	---------------------------------------------------
AN2868.1  	EVVRRWGGEGLLSAVPPKKEVHEAREDILESIEEMKFYKEQLFGASKLANK
          	.     ....  :: .....  .: ..  .: .. .  ...  .::. :..
```
